# Biotin and high-sensitivity cardiac troponin T assay

**DOI:** 10.11613/BM.2018.030901

**Published:** 2018-10-15

**Authors:** Aurélien Schrapp, François Fraissinet, Charles Hervouet, Hélène Girot, Valéry Brunel

**Affiliations:** 1 General Biochemistry Department, Rouen University Hospital, Rouen, France; 2 Pharmacy Department, Rouen University Hospital, Rouen, France

**Keywords:** troponin T, biotin, multiple sclerosis, preanalytical phase

## Abstract

**Introduction:**

The high-sensitivity cardiac troponin T assay of Roche Diagnostics is known to have interference with high concentrations of biotin as this assay uses biotin-streptavidin binding as detection method. As studies so far have not shown if different biotin concentrations could have diverse influence on various troponin concentrations and whether interference could be removed by available protocol within corresponding turnaround time we aimed to investigate it.

**Materials and methods:**

Plasma samples were spiked with different concentration of biotin solution. Troponin T concentrations were tested on a Roche Cobas® 8000 module 602 analyser. Final concentrations of biotin and troponin T were 50, 100, 500 and 1000 μg/L and 18, 59, 201 and 6423 ng/L, respectively. Impact of different incubation times following biotin neutralization protocol described by Piketty *et al.* was also tested.

**Results:**

We observed a mean of negative biases of 24, 56, 97, and 98% of the troponin T expected value at biotin concentrations of 50, 100, 500, 1000 μg/L. Neutralization protocol was applied on the sample with initial concentration of TnT of 59 ng/L at a biotin concentration of 1000 μg/L. Same results across different incubation times from 60 to 0 minutes were obtained (mean value 56.8 ng/L, coefficient of variation of 1.31%). We demonstrated that neutralization process had a dilution effect of the troponin concentration (loss of 4.5% to 9.6% of initial troponin value).

**Conclusions:**

Biotin interference is not dependent of initial troponin value. Interference could be successfully neutralized within a time frame compatible with emergency but results still should be carefully interpreted due to possible dilution effect.

## Introduction

Commercial high-sensitivity cardiac troponin T assay (hs-cTnT, Roche Diagnostics, Mannheim, Germany), a chemiluminescence immunoassay, is generally robust and not much data about interferences on the test has been described. No significant effect on hs-cTnT was observed with bilirubin or turbidity. However, hs-cTnT is negatively influenced by haemolysis (*1*). This negative interference seems to be time dependent and can be managed by laboratories with systematic measurement of sample haemolysis. Interferences of heterophilic antibodies have also been described (*2*). Recently, Trambas *et al.* described a negative interference affecting the result of troponin T by using hs-cTnT assay due to concentration of biotin above physiological value (*3*). The interference caused by high concentration of biotin is likely to affect all immunoassays using the biotin-streptavidin complex binding as detection method. The impact of observed interference is dependent on the concentration of biotin and the tested parameter. In case of interference, assay results are thus increased for competitive methods and decreased for sandwich methods (*3*). Biotin is a water-soluble vitamin, involved in many metabolic processes, with recommended daily intake of 30–75 μg. In the last decade, very high-doses of biotin (300 mg/day) have been shown to have a beneficial effect in patients suffering from progressive multiple sclerosis (*4*). Recently, interference removal protocols have been proposed to eliminate biotin in samples (*5*, *6*). Such protocols using pre-incubation with streptavidin-coated magnetic particles contained in Elecsys® immunoassay kits are easy to implement in laboratory workflow. Studies conducted so far recommend an incubation time of neutralization protocol from 45 minutes to 1 hour which can be a concern in terms of turnaround times for an emergency biomarker (*5*, *6*). At Rouen University Hospital, we perform approximately 50,000 hs-cTnT assays each year and up to 40% are for the emergency department. The aim of the study was to investigate if different biotin concentrations could have diverse influence on various troponin concentrations and whether neutralization of biotin interference could be removed by available protocol within corresponding turnaround time.

## Materials and methods

In order to conduct analysis residual plasma samples from patients admitted to hospital were used for spiking and depletion experiments. Blood was collected on BD Vacutainer® Lithium Heparin (Becton, Dickinson and Company, Franklin Lakes, NJ, USA) and plasma samples were obtained by centrifugation (1700xg, 10 min). Following routine analysis residual samples with concentration close to the 99th percentile of healthy controls (14 ng/L), to the cut-off proposed by the European Society of Cardiology (52 ng/L) which has a positive predictive value for myocardial infarction of 75 to 80%, to a positive free value (200 ng/L) and with a very high concentration of troponin T (5000 ng/L) were selected, anonymized, pooled, aliquoted (900 µL) and used in further analysis (*7*). Biotin was obtained from intramuscular injection solution (Biotin Bayer® 5 mg/mL, Bayer, Lyon, France). This solution was diluted in deionized water (1:500; 1:1000; 1:5000; 1:10,000) and 100 µL of these dilutions were added to 900 µL of each plasma sample to reach a final biotin concentration of 50, 100, 500 and 1000 µg/L, for each concentration of troponin. Control plasma samples were spiked with an equal volume of deionized water to account for possible matrix effects. The hs-cTnT assay was performed once *per* aliquot. Then neutralization of the interference was studied on the samples spiked with biotin. The neutralization method was performed as described by Piketty *et al*. (*8*). We used magnetic microparticles coated with streptavidin (0.72 mg/mL) contained in Elecsys® immunoassay kits. Reagent containing microparticles coated with streptavidin (1.5 mL) was centrifuged (1700xg, 10 min), and the supernatant was discarded. This neutralization consists of the incubation of the plasma (300 µL) with the microparticles coated with streptavidin (1.08 mg). The mixture was centrifuged 10 minutes at speed of 1700xg, as it is recommended by French Society of Clinical Biology for daily use in the laboratories, instead of 3000xg as it was suggested in original protocol (*9*). Plasma was removed to be tested. The bias expressed as a percentage was obtained with the formula: ([troponin concentration result in sample spiked with biotin] - [troponin concentration result in control sample]) / [troponin concentration result in control sample] x 100. If the measured value of troponin was below the limit of blank (3 ng/L), the calculation was done by replacing troponin concentration result in sample spiked with biotin by 3 ng/L and presenting the result as a minimum of bias. These results were excluded to calculate mean of biases. In addition, different incubation times of 60, 45, 30, 15, and 0 minutes of streptavidin-coated magnetic particles and plasma mixture after a 10-second vortex in sample with concentrations of troponin and biotin of 59 ng/L and 1000 µg/L, respectively, was investigated. Finally, we evaluated the dilution operated by the residual wet layer of magnetic microparticles used for the neutralization. The process of neutralization, without incubation time, was performed in five technical replicates on the sample free from biotin with the lowest concentrations of troponin 18, 59 and 201 ng/L. The hs-cTnT assay was performed once per neutralization procedure. Results of troponin are expressed in ng/L. Bias, mean of biases and coefficient of variation were calculated by the Microsoft Excel software. Assays for hs-cTnT were performed on a Roche Cobas® 8000 e602 analyser (Roche Diagnostics, Mannheim, Germany) and following the method provided by the manufacturer.

## Results

Final concentrations of troponin T in control samples were measured at 18, 59, 201, and 6423 ng/L. We observed a mean negative bias of initial troponin concentration of 23.5%, 55.8%, 96.7%, 98.2% at 50, 100, 500, 1000 µg/L biotin concentrations, respectively (Table 1). It should be noted that biotin overloads of 500 and 1000 μg/L decreased the initial troponin values of 18 and 59 ng/mL lower than the limit of blank (3 ng/L) making it impossible to calculate bias value. Considering biotin interference neutralization protocol, obtained results showed that there is no impact of different incubation times on results of troponin concentrations. A complete suppression of interference was obtained following mixing the sample and microparticles without further incubation. Results are presented in Table 2. The reiteration of neutralization process showed a systematic dilution from 4.5% to 9.6% of initial troponin value (Table 3).

**Table 1:** Effect of biotin on troponin T concentrations measured by hs-cTnT assay

**Troponin T concentration,** **ng/L**	**Biotin concentration, µg/L**			
	**50**	**100**	**500**	**1000**
18	25.4%	59.4%	> 82.9%^*,‡^	> 82.9%^*,‡^
59	24.7%	55.8%	> 94.8%^*,‡^	> 94.8%^*,‡^
201	21.6%	55.3%	96.4%^‡^	98.0%^†,‡^
6423	22.4%	52.8%	97.0%	98.5%
**Mean of biases**	23.5%	55.8%	96.7%	98.2%
Results are shown as negative biases in percentage before applying neutralization protocol. Values below limit of blank were not taken into account for calculation of the mean of biases. *Value below the limit of blank (3 ng/L). ^†^Value below the limit of detection (5 ng/L). ^‡^Value below the 99th percentile (14 ng/L).				

**Table 2:** Impact of different incubation times applied in biotin interference neutralization protocol on hs-cTnT

**Time, min**	0	15	30	45	60
**hs-cTnT, ng/L**	57	56	58	57	56
**hs-cTnT, ng/L; CV**	56.8; 1.31%				
hs-cTnT - high sensitive troponin T. CV - coefficient of variation. The acceptable limit defined by the manufacturer for coefficient of variation is 6%.					

**Table 3:** Impact of dilution effect on hs-cTnT result after biotin interference neutralization protocol

**hs-cTnT, ng/L**	**Mean hs-cTnT following biotin neutralization, ng/L (N = 5)**	**Mean of biases,** **% (N = 5)**
18	16.8	- 6.5%
59	56.3	- 4.5%
201	181.8	- 9.6%
hs-cTnT - high sensitive troponin T. Troponin T concentration is presented as mean of the value obtained after neutralization protocol repeat five times. Bias was calculated with the formula: [(Troponin T concentration following biotin neutralization) - (Initial troponin T concentration)] / (Initial troponin T concentration) x 100.		

## Discussion

Our study shows that hs-cTnT assay results are decreased by increasing biotin concentrations on various troponin concentrations. These interferences are not influenced by the initial troponin concentration. Indeed, we observed close negative biases at the same concentrations of biotin regardless of the initial value of troponin. High plasma concentrations of biotin from 500 to 1000 µg/L biotin, which are found in patients treated for multiple sclerosis, decreased the true value by more than 96% (*5*). These results are consistent with those of Trambas *et al.* who had observed a greater than 95% decrease in the troponin value at a biotin concentration of 500 µg/L (*3*). However, the risk generated by this kind of intake is not very complex to manage by the laboratory. In fact, in France, this medicine has a temporary authorization of use which implies that all the deliveries are traced by the central pharmacy of the establishment. The package leaflet informs of possible interference on many biological assays and moreover, at our institution, this information is systematically provided orally to the patient with each drug delivery. Central pharmacy regularly sends us the list of patients that we referenced in our laboratory software. This allows us to quickly identify samples requiring pre-treatment. In this study, assuming that the dissociation constant of biotin to streptavidin is very low (10^−14^ mol/L), we demonstrated that proposed incubation time of 1 hour in biotin interference neutralization protocol is not necessary. Thus, turnaround time of troponin could be improved if neutralization protocol should be applied. We confirmed interference removal via neutralization method and absence of difference in case of suppression of the incubation time in three specimens from two patients with acute coronary syndrome who were treated for multiple sclerosis by high dose of biotin.

Trambas *et al*. demonstrated, almost 10% and 20% negative bias at 15.6 µg/L and 31.3 µg/L of biotin, respectively (*3*). This dose response curve of biotin interference was evaluated at only one concentration of troponin close to 650 ng/L for hs-cTnT assay which has a measurement range of 3 to 10,000 ng/L. In addition, this value is not the most frequent or clinically interesting value. In fact, median of the troponin values measured in the emergency department of Rouen University Hospital is 19 ng/L. In our work, we studied the interference profile of four different concentrations of troponin and our results are similar to those of Trambas *et al*. for moderately high values of biotin. In fact, 45% of negative bias was observed on dose response curve at 60 µg/L of biotin (*3*). We observed at troponin concentrations of 18, 59, 201 and 6423 ng/L a mean bias of 23.5% and 55.8% at 50 µg/L and 100 µg/L of biotin. We demonstrated that the observed bias was independent of the initial troponin concentration. This plasma concentration of 100 µg/L can be found in patients one hour after oral administration of 10 mg of biotin, a dosage close to that recommended in a context of alopecia (15 mg/day) which does not require medical prescription (*9*). The risk generated by this kind of intake is much more complex to manage by the laboratory and its frequency is difficult to assay. Indeed, it is complicated for laboratories and for emergency practitioners to obtain information on drugs taken as self-medication. The risk induced is to give a false result lower than the 99th percentile, but also to simulate kinetics that do not exist because of the decay of blood biotin and the associated removal of interference. We recommend that laboratories alert physicians to this interference and collect all information regarding biotin intake. We also proved that this inactivation can be almost immediate and an incubation time can be avoided. For an emergency biomarker where the turnaround time highly impacts the management of the patient, this is essential. However, according to our results this pre-treatment can decrease the troponin value by 10% and this non-automated manipulation can alter the analytical performance, especially by increasing the analytical coefficient of variation. We did not study this decrease in troponin value in the very high concentration troponin aliquot. Indeed, the existence of a significant bias in these extreme values will not change the patient’s care in emergency department. If myocardial infarction is suspected, a troponin T assay should be performed at initial admission of the patient followed by a second assay at one hour later, known as H0/H1 protocol by the European Society of Cardiology (*7*). This protocol requires a high level of analytical quality since a variation of 5 ng/L between the two samples guides the physician to the diagnosis of myocardial infarction (*7*). This protocol should be avoided for neutralized samples. The H0/H3 protocol (second assay at 3 hours later) whose 30% variation between the two values is suggestive of acute coronary syndrome could still be used with caution.
